# Association between distance to community health care facilities and COVID-19–related mortality across U.S. counties in the COVID-19–vaccine era

**DOI:** 10.1186/s13104-023-06366-3

**Published:** 2023-06-05

**Authors:** Wenxi Huang, Inmaculada Hernandez, Shangbin Tang, Sean Dickson, Lucas A. Berenbrok, Jingchuan Guo

**Affiliations:** 1 Department of Pharmaceutical Outcomes and Policy, University of Florida College of Pharmacy, Gainesville, FL USA; 2 Skaggs School of Pharmacy and Pharmaceutical Sciences, University of California San Diego, San Diego, CA USA; 3 West Health Policy Center, Washington, DC USA; 4 Department of Pharmacy and Therapeutics, University of Pittsburgh School of Pharmacy, Pittsburgh, PA USA

**Keywords:** COVID-19 mortality, Social determinant of health, Health disparities, Geographic proximity, Healthcare access, Racial disparities

## Abstract

**Objective:**

COVID-19 has caused tremendous damage to U.S. public health, but COVID vaccines can effectively reduce the risk of COVID-19 infections and related mortality. Our study aimed to quantify the association between proximity to a community healthcare facility and COVID-19 related mortality after COVID vaccines became publicly available and explore how this association varied across racial and ethnic groups.

**Results:**

Residents living farther from a facility had higher COVID-19–related mortality across U.S. counties. This increased mortality incidence associated with longer distances was particularly pronounced in counties with higher proportions of Black and Hispanic populations.

## Introduction

The COVID-19 pandemic has had a devastating public health impact, with more than 900,000 deaths in the U.S. as of February 2022 [1]. At the early stage of COVID-19 breakout, racial and ethnic minority groups are shown to be significantly associated with an increased risk of COVID-19 infections and related mortality [2]. Vaccines against SARS-CoV-2 effectively reduce the risks of COVID-19 and related mortality [3–6]. However, the vaccine distribution plans were created with limited health equity components being considered [7]. Previous study has shown that racial and ethnic minority groups are less likely to live near healthcare facilities than their White counterparts, and this may also impact the equity of access to COVID-19 vaccines [8].

Previous studies reported disparities in geospatial access to healthcare facilities during the COVID-19 pandemic, such as the identification of vaccine deserts [9] and measuring accessibility COVID-19 health care resource including testing [10–12]. However, research gaps exist regarding to what extent the geospatial access to health care impacts COVID-19 related health outcomes. Therefore, we aimed to evaluate whether spatial access to community health care facilities, i.e., facilities that have the potential to serve vaccine administration, would have an impact on COVID-19 mortality. Here, we estimated the association between new COVID-19–related mortality and proximity to a community health care facility eligible to administer vaccines after they became widely available in the U.S. Additionally, we examined how this association varied across racial and ethnic groups.

## Methods

This study was considered exempt from formal review by the University of Florida, Gainesville, Institutional Review Board, and followed the STROBE reporting guideline. We geocoded all U.S. community pharmacies, federally qualified health centers, rural health clinics, and hospital outpatient departments registered as eligible to administer vaccines and calculated the health care facility density based on a prior study [8]. For a 1% sample of a U.S. synthetic population of 2,982,544 individuals, we computed the driving distance to the closest facility using ArcGIS Network Analyst and the National Transportation Dataset [13]. We calculated the median distance to the nearest facility for all individuals sampled within each U.S. county. We extracted county-level information on sociodemographic characteristics from the University of Wisconsin’s County Health Rankings and Roadmaps data, [14] including median household income, racial and ethnicity composition (i.e., the percentage of the county population that was non-Hispanic Black and Hispanic, receptively), and percentage of the county population that were college educated, female, unemployed, and uninsured; all of these variables were continuous variables in the original form and we dichotomized these covariates using a cut-off of the top 20% and bottom 80%. We classified U.S. counties as rural or urban based on the U.S. Department of Agriculture’s rural-urban continuum codes.

The study outcome was county-level COVID-19–related mortality [1] from May 1 to November 30, 2021. We calculated incidence rate ratios (IRRs) using Poisson regressions at the county-level to examine the association between the median distance to the nearest facility and COVID-19–related mortality, accounting for the county population using an offset variable, and controlling for county-level rurality, health care facility density, COVID-19 infection rate [1], and sociodemographic characteristics (i.e., proportion with college education, female, unemployed, and uninsured, and median household income). We conducted subgroup analyses by racial and ethnic compositions.

## Results

**Fig. 1:**
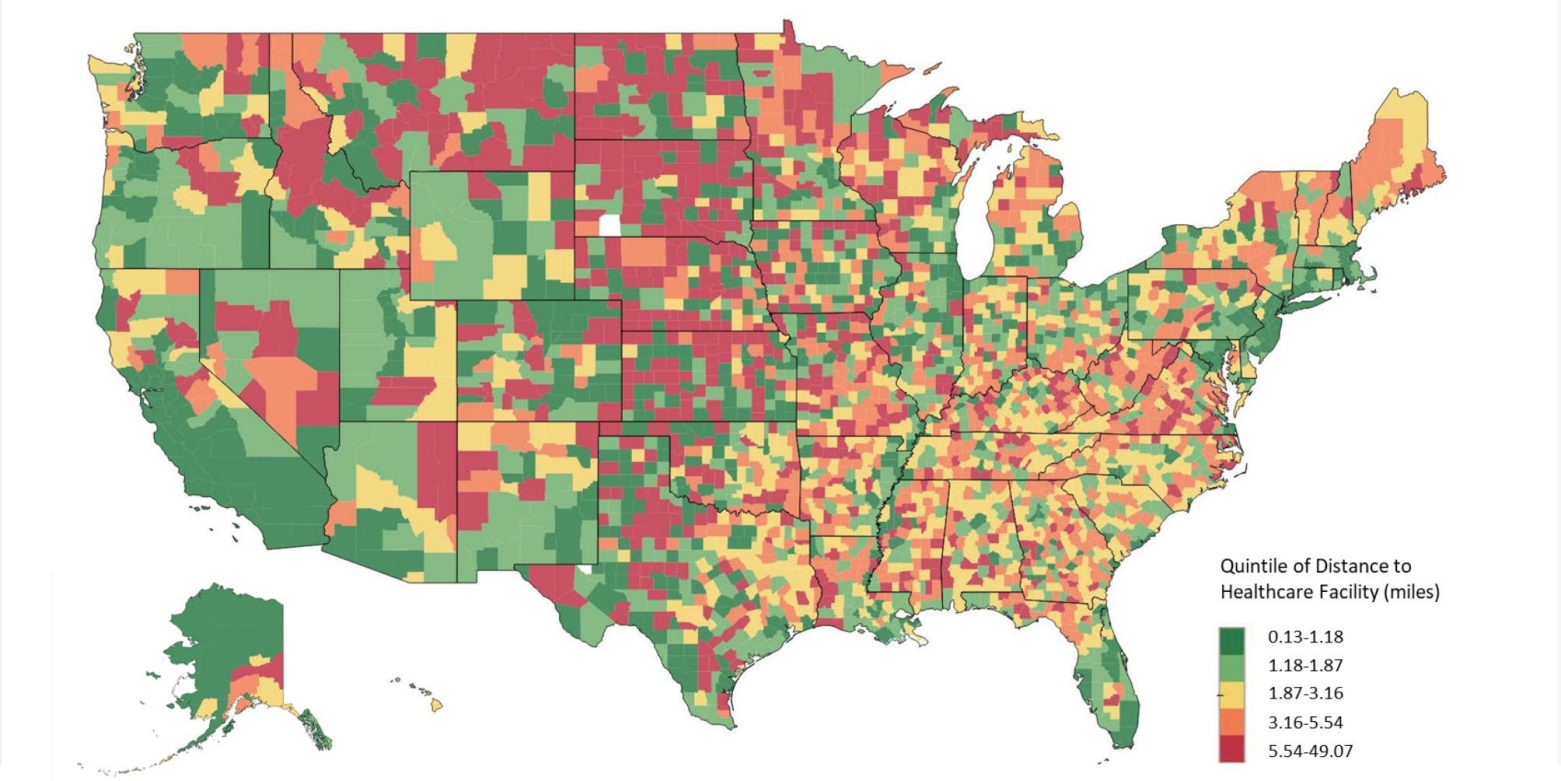
Quintile Distribution of the Median Distance in Miles to the Nearest Community Health Care Facility Across U.S. Counties

This study included 2,932 counties in the US states (excluding territories). In total, 23.8% of counties had a median distance of more than 5 miles to the nearest community health care facility. Figure 1 shows the geographic variation in proximity to community health care facilities across U.S. counties. Counties in the top 20% of median distance to the nearest facility (i.e., median distance > 5.5 miles) were concentrated in Missouri, Texas, and Minnesota.

Counties with longer distance to healthcare facility (counties in the top 20% of the median distance to the nearest facilities) compared to the remaining counties has less female, less non-Hispanic Black and Hispanic population, lower household income, less college educated population and more unemployed and uninsured population. (Table 1)

**Table 1:** Characteristics of counties have long distance to healthcare facility (in the top 20% of the median distance to the nearest facilities) compared to the remaining counties

Variable^3^	Overall, N = 2,932^1^	Short Distance to Healthcare Facility, N = 2,345^1^	Long Distance to Healthcare Facility, N = 587^1^	P-Value^2^
Female, %	50.4 (49.5, 51.1)	50.5 (49.7, 51.2)	49.9 (49.0, 50.5)	< 0.001
High Non-Hispanic Black^3^	590 (20%)	496 (21%)	94 (16%)	0.005
High Hispanic^3^	586 (20%)	537 (23%)	49 (8.3%)	< 0.001
High Household Income^3^	589 (20%)	529 (23%)	60 (10%)	< 0.001
High College Educated^3^	583 (20%)	491 (21%)	92 (16%)	0.004
High Unemployed^3^	590 (20%)	450 (19%)	140 (24%)	0.012
High Uninsured^3^	586 (20%)	448 (19%)	138 (24%)	0.017
High Health Care Density^3^	587 (20%)	481 (21%)	106 (18%)	0.2
High Covid-19 Infection Rate^3^	588 (20%)	474 (20%)	114 (19%)	0.7
High Mortality^3^	590 (20%)	459 (20%)	131 (22%)	0.14

^1^Median (IQR); n (%)

^2^Pearson’s Chi-squared test

^3^High stands for counties in the top 20%

**Fig. 2:**
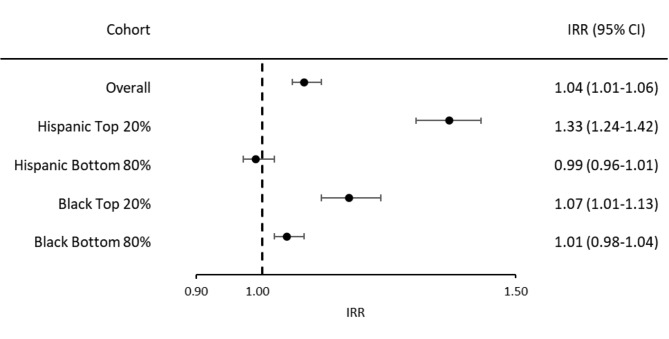
Adjusted incidence rate ratios (IRRs) of COVID-19–related mortality for counties in the highest quintile (i.e., top 20% of county median distance to nearest community health care facility) vs. the remain counties. Model adjusted for county-level information on rurality classification, commnity health care facility density, COVID-19 infection rate, median household income, racial and ethnicity composition, and proportions of population that were college educated, female, unemployed, and uninsured. Categories of race and ethnicity included Asian, Black, Hispanic, and White residents as well as “other,” which comprised American Indian and Alaska Native, Native Hawaiin and Pacific Islander persons

Distance to a health care facility was positively associated with COVID-19–related mortality (IRR of counties in the highest quintile [top 20%] of county median distance to nearest facility vs. the remain counties, 1.67; 95% CI, 1.64‒1.71) in the unadjusted model; the effect size was attenuated in the fully adjusted model but remained statistical significance, 1.04; 95% CI, 1.01‒1.06). That is, after controlling for sociodemographic characteristics and COVID-19 infection rate, COVID-19–related mortality after vaccine availability was 4% higher for counties with a median distance farther vs. closer than 5.5 miles (Fig. 2).

The association between the median distance to a facility and COVID-19–related mortality was more pronounced in counties in the top 20% of Hispanic composition (IRR, 1.33; 95% CI, 1.24‒1.42) and the top 20% of Black composition (IRR, 1.07; 95% CI, 1.01‒1.13) (Fig. 2).

## Discussion

To our knowledge, this is the first nationwide study to examine the county-level association of geographic proximity to community health care facilities with COVID mortality in the US. This study addressed geographic proximity to community health care facilities as a social determinant of health (SDoH) that presents additional barriers to accessible care and improved health outcomes. Residents in counties with a greater median distance from community health care facilities experienced higher mortality related to COVID-19 across U.S. counties. The increase in mortality risk associated with longer distances was particularly pronounced in counties with higher proportions of Black or Hispanic residents. Approximately 80% of individuals residing in areas of concentrated poverty are Black or Hispanic, as indicated by national statistics [15]. Compared to earlier phases of COVID-19 deaths in 2020, where an increase in the percentage of the Black population at the county level was linked to a greater rise in COVID-19 deaths per 100,000 population than the Hispanic population, [2] our study showed a higher risk of COVID-19 mortality in counties with concentrated Hispanic populations compared to those with concentrated Black populations.

The substantial health disparities observed among the Hispanic population may be attributed to a range of factors, including differential loss of health insurance, suboptimal quality of care, inequitable distribution of limited testing and medical interventions, disparities in digital access, food and housing insecurity, as well as work-related exposures [16–18]. For example, Hispanic individuals usually lack adequate insurance coverage, which hinders their access to preventive healthcare [16].

Additionally, a recent systematic review suggested that studies examining travel distance and health outcomes were limited and the results were mixed; neighborhood level socioeconomic status appeared to be an important confounding factor that the travel distance-health outcome association was attenuated after adjusting for neighborhood level socioeconomic status [19]. In our study, we observed a significant and positive association between travel distance to community health care facilities and COVID-19 related mortality even after adjusting for several aspects of neighborhood demographic and socioeconomic factors, such as education attainment, unemployment, and insurance status. Our results support spatial distance to healthcare as a structural social risk factor in addition to conventional SDoH. Importantly, we identified significant interactions between spatial distance to healthcare and racial-ethnic composition in the association of COVID-19 related mortality: the spatial distance to community health care facilities became a stronger risk factor for COVID-19 mortality in counties with higher Black and Hispanic compositions. We believe that our findings have important implications that spatial access to healthcare might be a structural but modifiable factor to address structural inequities across racial and ethnic groups.

In conclusion, our findings highlight persistent and structural inequities in access to existing community health care infrastructure and have broader implications beyond the COVID-19 pandemic. In our study, a longer geographic distance to community healthcare facilities was associated with a higher risk of COVID-19 mortality. More importantly, we identified that the impact of geographic access to healthcare on COVID-19 mortality was more profound in counties with the highest Black and Hispanic composition. Our results are critical as it reveals geopathic proximity to community healthcare as a potential underlying factor of structural inequities associated with race and ethnicity. As such, it is crucial to design policy and intervention programs that focus on under-resourced and marginalized communities to address these inequities.

Several interventions and programs can be implemented to improve access to healthcare in under-resourced communities. One approach is to establish high-quality community health care facilities that provide comprehensive services, including preventive care, chronic disease management, and behavioral health services [20]. Another approach is to develop community health worker programs that provide education and support to individuals and families in underserved communities [21]. Expanding public transportation or adding mobile health clinics can also improve access to healthcare facilities for individuals who rely on public transportation [22, 23]. Strengthening community partnerships between healthcare providers, community organizations, and local governments can help identify and address the specific needs of underserved communities [24]. These interventions and programs can help reduce the impact of structural inequities in access to healthcare and improve health outcomes for vulnerable populations.

## Limitations

This study had limitations. We calculated access to community health care facilities where COVID-19 vaccines may be administered and not actual COVID-19 vaccination locations. However, investigating existing community health care facilities addresses the importance of developing community health care infrastructure in response to public health crises. In addition, Hispanic individuals are heterogeneous with racial and geographic diversity which cannot be analyzed using our current data.

## Data Availability

The data presented in this study are available on request from the corresponding author The data presented in this study are available on request from the corresponding author.
